# *Corynebacterium nuruki*: A novel pathogen in human catheter-related bacteremia

**DOI:** 10.1016/j.nmni.2025.101574

**Published:** 2025-03-04

**Authors:** Pablo Fernández-Palacios, Ramón Vela-Fernández, Francisco Javier Capote, Estefanía Jurado-Tarifa, Manuel A. Rodríguez-Iglesias, Fátima Galán-Sánchez

**Affiliations:** a Servicio de Microbiología, Hospital Universitario Puerta del Mar, Cádiz, Spain; b Servicio de Hematología, Hospital Universitario Puerta del Mar, Cádiz, Spain; c Instituto de Investigación e Innovación Biomédica de Cádiz (INIBICA), Hospital Universitario Puerta del Mar, Cádiz, Spain; d Departamento de Biomedicina, Biotecnología y Salud Pública, Universidad de Cádiz, Cádiz, Spain

**Keywords:** *Corynebacterium nuruki*, Catheter-related bacteremia, Whole genome sequencing, 16S rRNA sequencing

## Abstract

A 72-year-old with lymphoma developed catheter-related bacteremia caused by *Corynebacterium nuruki,* a microorganism previously unlinked to human infections. Diagnosis included MALDI-TOF and genomic sequencing. Bacteremia was treated with vancomycin and the catheter was replaced. This case introduces *C. nuruki* as a potential pathogen in immunocompromised patients.

## 1 Case presentation

A 72-year-old patient with marginal lymphoma progressing after several lines of treatment was brought by his family to the emergency department of a hospital in southern Spain due to deteriorating general condition, anorexia, fever and confusion. The patient has not travelled outside their region recently. The patient had no cardiovascular, respiratory, genitourinary or gastrointestinal symptoms. Ischemic cerebrovascular disease was ruled out by computerized tomography scan. Blood test showed an increase in C-reactive protein (211.0 mg/L), LDH (256.0 U/L) and procalcitonin (16.84 ng/mL), which could indicate an infectious etiology.

The patient carried a port-a-cath catheter that had never been used, with signs of external infection. Admission to the haematology ward was decided and treatment was started with piperacillin-tazobactam. Four blood samples (10 mL each) were simultaneously drawn from the suspect device and a peripheral vein, and were placed into 2 pair of blood culture bottles (BD Bactec® Plus Aerobic/F and Plus Anaerobic/F), to rule out catheter-related bacteremia. The four samples were processed in the BD Bactec® Fx System (Beckton, Dickinson and Company, Franklin Lakes, NJ).

**Fig. 1:**
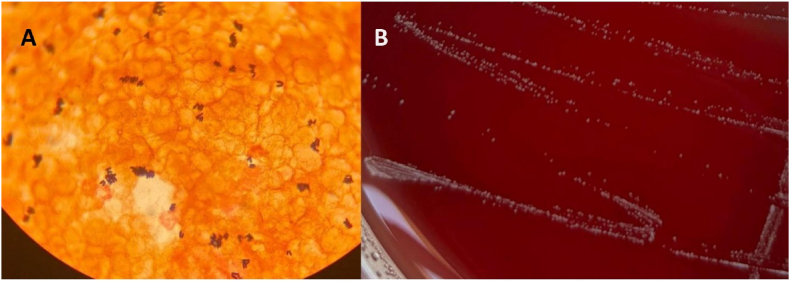
(A) Gram stain from blood culture, Gram-positive bacilli. (B) Bacterial growth (2 days) in anaerobic atmosphere at 37 °C in BD Agar Columbia BBL™ Sheep Blood.

After 23 and 27 hours of incubation, the two aerobic bottles from the catheter and venipuncture flagged positive, respectively. Gram staining from both bottles reveals gram-positive, palisade-shaped bacilli suggestive of *Corynebacterium* spp. (Fig. 1A). Small, white and punctate colonies were observed after 24 hours of incubation in blood agar (anaerobic atmosphere at 37 °C in BD Agar Columbia BBL™ with 5 % Sheep Blood) (Fig. 1B) and chocolate agar (5–7% CO_2_ atmosphere at 37 °C in BD BBL™).

Identification using MALDI-TOF (Bruker Daltonics, Bremen, Germany) directly from the blood culture yielded *Corynebacterium amycolatum* as the first proposed identification with a low score of 1.32, and repetition from colonies did not produce any result. Based on Gram staining observations, the isolate was considered as an unidentified species of *Corynebacterium*. Treatment with piperacillin-tazobactam was changed to meropenem and vancomycin two days after.

Antimicrobial susceptibility testing was performed using the disk diffusion method according to the European Committee on Antimicrobial Susceptibility Testing (EUCAST) recommendations. The antibiotics tested and their clinical categories according to EUCAST guidelines (v.13.0) were: penicillin (susceptible increased exposure), clindamycin (resistant), vancomycin (susceptible), ciprofloxacin (susceptible increased exposure), and rifampicin (susceptible). Treatment was not modified as the isolate was susceptible to vancomycin. Through the partial sequencing of the 16S ribosomal RNA gene, the isolates were identified as *Corynebacterium nuruki* (*C. nuruki* HUPM 1) with 100 % identity using BLAST (http://blast.ncbi.nlm.nih.gov). The port-a-cath catheter was not removed due to severe coagulopathy, although the patient improved and was discharged two weeks later.

Ten days later he was readmitted with similar symptoms to the initial admission. Piperacillin-tazobactam was prescribed and four samples of blood were taken again to rule out catheter-related bacteremia**.** After 53 hours of incubation, the aerobic bottle from the catheter flagged positive and an isolate with the same micro-and macroscopic morphology than *C. nuruki* was recovered. On this second admission, the medical staff decided to change the port-a-cath catheter for a peripherally inserted central catheter (PICC). Port-a-cath catheter culture was not performed. The patient improved and was discharged one month after admission.

Partial sequencing of the 16S ribosomal RNA gene and whole genome sequencing (WGS) of the second isolate of *Corynebacterium* spp (*C. nuruki* HUPM 2) were performed. The isolate was identified as *C. nuruki* by both methods.

## 2 Discussion

Non-Diphtheriae *Corynebacterium* species are composed of gram-positive, non-sporulating, non-motile, anaerobe facultative and pleomorphic bacteria. In the microscope slides are shown in a palisade or Chinese letters (Fig. 1A) [1]. Usually, they are considered contaminants in clinical samples due to their presence as normal skin and mucosal flora. However, they may have clinical relevance, especially in immunocompromised patients and/or patients with medical devices or catheters, where they may cause true bacteremia [2].

Diagnosis of infections caused by species of *Corynebacterium* is based on culture of the corresponding samples and subsequent identification by MALDI-TOF. *Corynebacterium* is characterized by small white or grey colonies that usually grow on enriched media such as Chocolate agar and Blood agar within 24–48 hours of incubation. (Fig. 1B). Some species need longer incubation time, lipophilic media and may adopt different morphologies in the culture media [3].

The use of mass spectrometry has made it possible to discover the first cases of infections in humans caused by microorganisms that were common in animal and the environment samples. Even so, mass spectrometry still has limitations as it does not contain in its database all existing microorganisms, as it is the case with *C. nuruki*. In addition, there are *Corynebacterium* species that MALDI-TOF is unable to discriminate because of the high similarity between them, such as *C*. *aurimucosum* from *C*. *minutissimum* and *C*. *minutissimum* from *C*. *singular* [4].

Before the introduction of MALDI-TOF in the microbiology diagnosis, biochemical methods were routinely used for the identification of microorganisms. The main disadvantage of these methods for the identification of *Corynebacterium* spp. is the difficulty of achieving species-level identification and their slowness. Molecular methods, such as the amplification and Sanger sequencing of partial 16S ribosomal RNA (∼540 bp), achieve better results than biochemical methods but with a higher cost [5]. However, it should be used when MALDI TOF identification does not provide a conclusive result, if it is clinically relevant, as in the case of a catheter-related bacteremia. In addition to the identification of microorganisms, partial sequencing of 16S ribosomal RNA is used for evolutionary and taxonomic studies [6].

When *Corynebacterium* spp. was isolated for the second time (*C. nuruki* HUPM 2), it was decided to perform again the 16S rRNA sequencing in order to confirm the identification, and to complete the study with WGS, since this species had not previously been linked to human infections. Bacterial DNA was extracted using an EZ1 Advanced XL following the manufacturer's instructions. WGS was performed using MiSeq (Illumina, Cambridge, UK) and Quast and Fastp were used to assess genome quality [7,8]. De novo assembly was performed with Qiagen CLC Genomics Workbench [9]. A neighbor-joining phylogenetic tree was constructed between *C. nuruki* HUPM 2 and isolates whose genomes are in the GenBank database (accessed August 26, 2024) using Prokka, Roary and FastTree [[10], [11], [12]]. Phylogenetic results and annotations were visualised using iTOL v.6 [13].

**Fig. 2:**
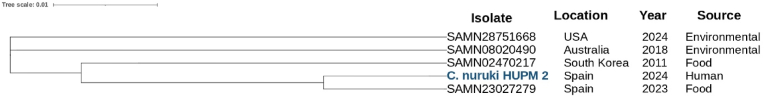
Phylogenetic analysis of *C. nuruki* HUPM 2 and *C. nuruki* isolates available in GenBank database (accessed August 26, 2024). A neighbor-joining phylogenetic tree was constructed to establish the phylogenetic relationships among the isolates based on core genes using Roary. The visualization of the phylogenetic tree and the annotations were done by iTOL v.6.

To date, there is very little information on *C. nuruki* and its ability to cause infections in humans. There are only four *C. nuruki* genomes in GenBank from environmental and animal samples (Fig. 2). The first isolation of *C. nuruki* was in 2011 in South Korea from a sample of nuruk, a traditional Korean fermentation starter (SAMN02470217) [14]. The phylogenetic tree shows that *C. nuruki* HUPM 2 shows the highest similarity to a sample from a Cabrales cheese from Asturias SAMN23027279 (Northern Spain). The two environmental samples are further apart in the tree (SAMN08020490, SAMN28751668. Asking the patient about any specific epidemiological links did not yield additional relevant information. *C. nuruki* is phylogenetically related to *C. variabile* found in cheese samples and to *C. terpenotabidum* isolated from soil samples [14]. The information obtained about the phylogenetic relationship of the isolates included in the tree is scarce and more studies are needed to provide more information on this microorganism. The isolate was identified as *C. nuruki* using the PubMLST and PathogenWatch platforms. The 16S rRNA sequence of *C. nuruki* HUPM 1 (PP440189) and the whole genome sequence of *C. nuruki* HUPM 2 (SAMN43227114) are available at GenBank.

Vancomycin is the antibiotic of choice for treating catheter-related bloodstream infections (CRBSI). Most CRBSIs are caused by gram-positive microorganisms, so vancomycin is a good choice because it is also effective against methicillin-resistant *Staphylococcus aureus* (MRSA) [15]. Due to the patient's clinical instability and immunological status, it was decided to treat with meropenem and vancomycin during the first admission. The duration of treatment for uncomplicated CRBI usually lasts between 7 and 14 days depending on the microorganism [15]. During the second admission, the patient was treated with piperacillin-tazobactam before the port-a-cath catheter removal. At the time of the first and second admissions, the patient was not considered to have CRBSI and this would explain why he was not treated with vancomycin from the beginning.

*Corynebacterium* spp. has been shown to have a high susceptibility to vancomycin [16]. The ResFinder and CARD platforms were used to search for resistance genes in the *C. nuruki* HUPM 2 genome, but no antimicrobial resistance genes were detected [17,18]. However, resistance mechanisms to fluoroquinolones (mutations in *gyrA* gene), rifampicin (mutations in *rpoB* gene), and beta-lactams (the *blaA* gene) have been described in other more resistant *Corynebacterium* species such as *C. jeikeium* or *C*. *urealyticum* ([19,20]).

The present case is of particular relevance because it is the first case of infection by *C. nuruki* described to date in humans. Although it is an apparently innocuous pathogen, this case opens the possibility of considerate *C. nuruki* as a new pathogen. The development of WGS allows for a better understanding of the genotypic characteristics of bacterial isolates and it establish epidemiological relationships with other isolates. *C. nuruki* had never before been associated with human infections and in our case, the origin of the port-a-cath infection could not be clarified.

## CRediT authorship contribution statement

**Pablo Fernández-Palacios:** Writing – review & editing, Writing – original draft, Visualization, Validation, Supervision, Software, Resources, Project administration, Methodology, Investigation, Funding acquisition, Formal analysis, Data curation, Conceptualization. **Ramón Vela-Fernández:** Methodology. **Francisco Javier Capote:** Methodology. **Estefanía Jurado-Tarifa:** Methodology. **Manuel A. Rodríguez-Iglesias:** Writing – review & editing, Writing – original draft. **Fátima Galán-Sánchez:** Writing – review & editing, Writing – original draft, Methodology, Conceptualization.

## Declaration of competing interest

The authors declare that they have no known competing financial interests or personal relationships that could have appeared to influence the work reported in this paper.
